# Skin Diseases-Related Enzymes: Mechanisms and Clinical Applications

**DOI:** 10.4061/2011/464507

**Published:** 2011-12-29

**Authors:** Yong-Doo Park, Jun-Mo Yang, Zhi-Rong Lü

**Affiliations:** ^1^Zhejiang Provincial Key Laboratory of Applied Enzymology, Yangtze Delta Region Institute of Tsinghua University, Jiaxing 314006, China; ^2^Department of Dermatology, Sungkyunkwan University School of Medicine, Samsung Medical Center, Seoul 135-710, Republic of Korea; ^3^Department of Environmental Health, School of Public Health and Tropical Medicine, Southern Medical University, Guangzhou 510515, China

There are higher numbers of known diseases of the skin than any other organ system due to the fact that skin is the first defense system against the external environment and it primarily responses to the various outer challenges. In this regard, studies on the skin diseases-associated enzymes are, therefore, important to understand the complex nature of defense mechanisms and various pathogeneses. 

In the present special issue, we focused on the several enzymes from the broad category of skin diseases-related enzymes that the authors mainly contributed the review and the research articles. We invited investigators to contribute to the several potential topics that expected to provide insights into the roles of enzymes in skin diseases, and, as the final outcome, six review articles that focused on matrix metalloproteinases, ADAMs family proteinases, autotaxin, AKT signaling, and manganese superoxide dismutase and four original research articles that are associated with pigmentation, psoriasis, and inflammation are published in the current special issue.

The review articles include the following:

- bifacial roles of matrix metalloproteinases (MMPs) was described: treatments for aging and cancer via inhibiting MMPs are paid attentions while stimulating MMPs is also applied to prevent wound scars and epidermal hyperproliferative diseases,
- the roles of a disintegrin and metalloproteinases (ADAMs) belonging to the zinc protease superfamily in a wide variety of skin diseases,
- mechanisms of autotoxin and its metabolite lysophosphatidic acid (LPA) in melanoma and potential treating strategy,
- therapeutic ideas for melanoma by regulating Akt3 and the related molecules in the pathway of AKT signaling,
- the roles of manganese superoxide dismutase in skin cancer and in inflammation,

and the original research articles describe the following:

- inhibition kinetics for tyrosinase, a core enzyme of pigmentation, accompanying with the computational docking simulation,
- enzymatic functional and structural studies for manganese superoxide dismutase that is well-known to associate with inflammation,
- bioinformatic analyses of probing therapeutic candidates for psoriasis by using PPI mappings and DB analyses,
- computational simulations and interactomics for the novel binding partners of creatine kinase.

In this special issue, with the efforts of the authors, several important diseases-associated enzymes have been newly understood, and the integrating studies between enzymology and computational simulations successfully revealed the factors associated with complex cutaneous diseases. This special issue also handles the several enzymes that are important for the clinical applications.

Publication of this special issue marks the part of the long journey of *Enzyme Research*, and I hope that this special issue serves a vehicle for the high-quality research of enzyme researchers in some respects.

